# Resolution of Visual Field Defect in Macroprolactinoma After Treatment With Cabergoline

**DOI:** 10.7759/cureus.25548

**Published:** 2022-05-31

**Authors:** Kimitaka Shibue, Momoko Yamakawa, Namiko Nishida, Akihiro Hamasaki

**Affiliations:** 1 Department of Endocrinology and Diabetes, Tazuke Kofukai Medical Research Institute, Kitano Hospital, Osaka, JPN; 2 Department of Ophthalmology, Tazuke Kofukai Medical Research Institute, Kitano Hospital, Osaka, JPN; 3 Department of Neurosurgery, Tazuke Kofukai Medical Research Institute, Kitano Hospital, Osaka, JPN

**Keywords:** pituitary adenoma size and eye, magnetic resonance imaging and pituitary adenoma, visual field defect, cabergoline, prolactinoma

## Abstract

We report the case of a 49-year-old woman presenting with amenorrhea and progressive visual field defect for one month. Endocrinological workup revealed a high concentration of serum prolactin, and magnetic resonance imaging (MRI) showed pituitary macroadenoma with extrasellar extension as well as compression of optic nerves. Treatment with a dopamine agonist, cabergoline, for eight weeks led to the resolution of the visual field defect accompanied by a rapid decrease in the serum prolactin level. Follow-up MRI three months after the initial diagnosis revealed alleviation of visible mechanical compression of the optic chiasm by the tumor. We considered that the absence of retinal nerve damage and prompt initiation of cabergoline contributed to the rapid recovery of the visual acuity.

## Introduction

Prolactinoma is one of the major secretory benign pituitary tumors, accounting for over half of all pituitary adenomas [1]. It is predominant in women (10 times more prevalent than in men) [2], but the sex difference disappears after menopause [3]. Prolactinoma is characterized by hyperprolactinemia due to the hypersecretion of prolactin from the tumor [4]. While macroprolactinomas are typically detected due to the presence of amenorrhea and galactorrhea, derived from their hormonal characteristics, they are also indicated by neurological symptoms, such as visual field defect and headache. Although visual field defect due to mechanical compression of the optic chiasm by the pituitary tumor can be transient, visual complications often become permanent if the diagnosis is delayed or retinal ganglion cells are damaged due to the long-term compression of the optic nerves [5].

Herein, we report a case of macroprolactinoma presented with amenorrhea and progressive visual disturbance and resolved by the prompt initiation of a dopamine agonist, cabergoline. This case indicates the significance of adequate evaluation of a mechanical complication caused by a pituitary tumor shortly after the initial diagnosis.

## Case presentation

A 49-year-old woman with hypertension presented to the Ophthalmology Department of our hospital with blurred vision left hemianopsia and difficulty in character recognition on reading persisting for one month. Although she had also been aware of amenorrhea over the previous months, she self-diagnosed it as the occurrence of natural menopause and did not seek consultation. She did not have a history of galactorrhea. A blood sample test revealed an elevated serum concentration of prolactin (4,452 ng/mL), consistent with prolactinoma (Table 1).

**Table 1:** Results of an endocrinological blood test after hospitalization.

Parameters	Value
ACTH (pg/mL)	27.2
hGH (ng/mL)	0.28
FreeT4 (ng/dL)	0.94
FreeT3 (pg/mL)	2.67
TSH (μIU/mL)	1.54
LH (mIU/mL)	1.56
FSH (mIU/mL)	11.5
Cortisol (μg/dL)	6.6
Prolactin (ng/dL)	4452
IGF-1 (ng/mL)	164

**Figure 1:**
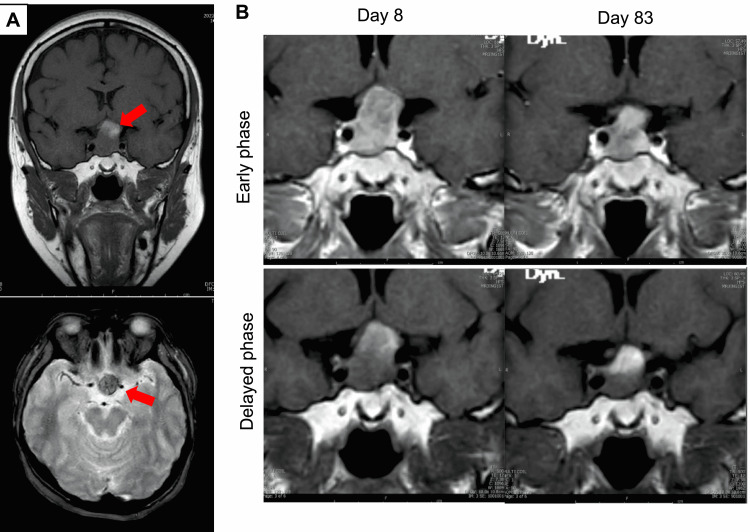
Magnetic resonance imaging (MRI) of the case. (A) Magnetic resonance (MR) image of the pituitary adenoma on the day of onset (Upper panel: coronal, T1W, Lower panel: horizontal, T2W. Red arrows indicate the pituitary adenoma). (B) Dynamic contrast-enhanced image of macroprolactinoma on day 8 (left panel) and day 83 (right panel). Early phase (upper panel) and delayed phase (lower panel) are shown.

A physical exam did not show eyelid ptosis or diplopia. Magnetic resonance imaging (MRI) conducted for the differential diagnosis of optic neuritis and pituitary adenoma revealed a solid pituitary tumor (28 mm) excluding the optic chiasm on the left side and invading the right cavernous sinus. The top of the tumor showed poor contrast, suggesting intratumor hemorrhage. The pituitary stalk deviated to the left side and there was a lesion showing early contrast on the left side of the tumor, suggesting compression of the frontal lobe of the pituitary (Figures 1A, 1B, left).

**Figure 2:**
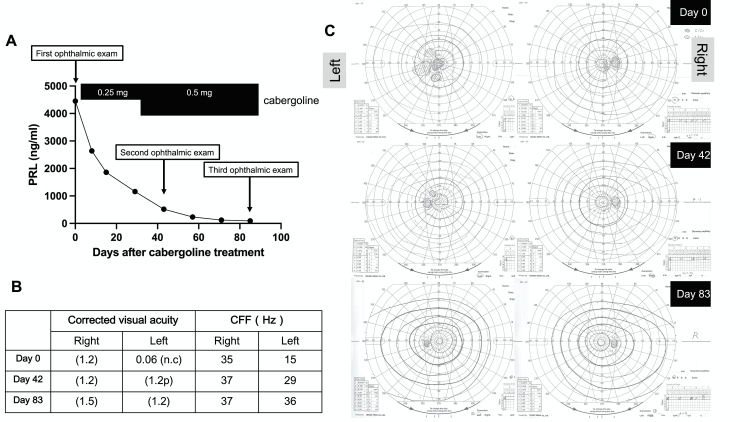
Clinical course of the macroprolactinoma. (A) Time-course of serum prolactin (PRL) concentration with ophthalmic examinations and the dose of cabergoline. (B) Time-dependent change of visual acuity and critical flicker frequency (CFF) test. (C) Time-dependent resolution of visual field defect. Visual field assessment was performed of both eyes on days 0, 42, and 83.

A visual test in the Ophthalmology Department demonstrated a severe drop in visual acuity and bitemporal hemianopia (Figure 2A). A critical flicker frequency (CFF) test showed a decreased value for the left eye, suggesting compression of the optic nerve (Figure 2B).

**Figure 3:**
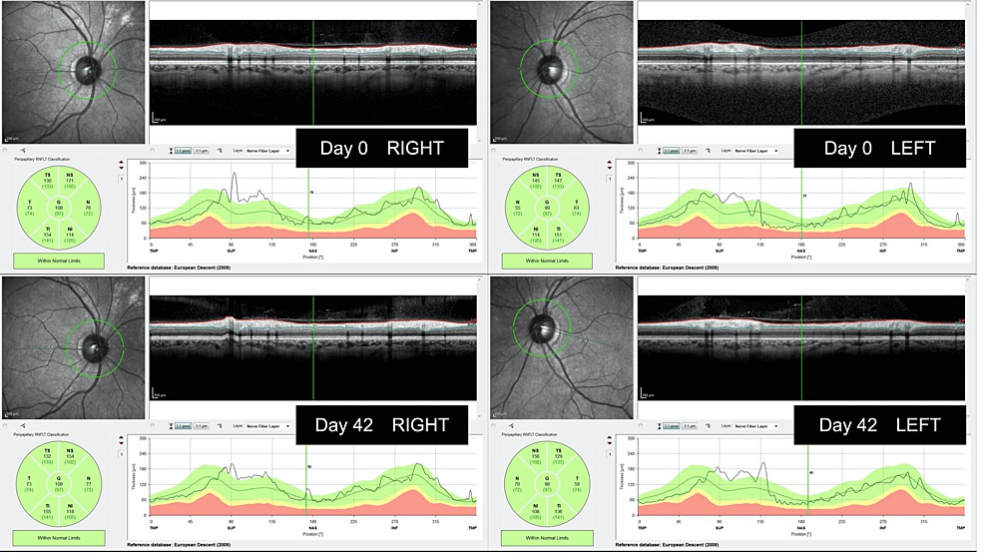
Pre- and post-treatment examination of the retinal nerve fiber layer thickness. Measurement of the retinal nerve fiber layer (RNFL) thickness with OCT on days 0 and 42. RNFL was measured using Spectralis OCT (Heidelberg Engineering GmbH, Heidelberg, Germany) and analyzed with Heidelberg eye explorer software version 1.9.14.0. Green circles around the optic disc were drawn to measure the peripapillary RNFL thickness.

On the other hand, optical coherence tomography (OCT) at the time of diagnosis as well as after cabergoline treatment revealed no retinal nerve fiber layer (RNFL) thinning (Figure 3). Collectively, we made a diagnosis of macroprolactinoma accompanied by the complication of acute-onset visual disturbance. Treatment with cabergoline, a dopamine agonist, was initiated at a dose of 0.25 mg once a week and increased to 0.5 mg once a week in the fourth week. The serum prolactin level showed a rapid decrease (Figure 2A) with a resolution of the right central hemianopia on day 42. While left hemianopia remained at that time, it was also resolved on day 83, together with the CFF value (Figures 2B, 2C). OCT performed on day 0 (pre-treatment) and day 42 (after the initiation of the cabergoline treatment) showed a preserved retinal nerve layer thickness throughout the clinical course (Figure 3). MRI taken on day 83 revealed tumor shrinkage (28 to 24 mm) (Figure 1B, right).

## Discussion

Common clinical features associated with macroprolactinoma are classified based on their causes: elevated circulating prolactin causes hypogonadism, amenorrhea, infertility, and galactorrhea while visual field defects and headache are due to mechanical compression derived from extrasellar extension of the tumor primarily by macroprolactinoma (>1 cm) or giant prolactinoma (> 4 cm) [6]. A large prolactinoma is associated with an increased risk of vision problems. A literature review revealed that >70% of patients with giant prolactinoma exhibited some type of visual disturbance [7]. From a mechanical perspective, while prolactinoma can extend in various directions, a suprasellar extension of the pituitary adenoma [8] is prone to causing visual field defects, being consistent with our case.

Cabergoline, a dopamine agonist, is the first-line medication for prolactinoma [9-11] providing therapeutic effects regardless of sex, age, or initial prolactin value [12]. Cabergoline use is also associated with a favorable prognosis regarding visual acuity [13,14].

Prognostic factors of visual acuity in prolactinoma patients are critical because they have direct effects on their long-term quality of life. Regarding pituitary adenoma treated with surgical resection, preoperative visual acuity is a prognostic factor for postoperative visual acuity and the visual field [15]. From an ophthalmologic viewpoint, abnormalities in the RNFL are useful to assess ocular damage caused by pituitary tumors [16].

While the preoperative RNFL thickness is considered to be a useful prognostic indicator of long-term visual recovery after trans-sphenoidal-approach surgery for pituitary adenoma [17], there are reported cases in which visual recovery was not associated with the RNFL thickness [18], and so this warrants further discussion. As a new parameter of OCT, analyzing the ganglion cell complex (GCC) [19] is considered to be a promising measurement strategy.

In our case, the patient consulted the hospital one month after the first subjective visual problem. Based on the preceding amenorrhea noted many months before the presentation and the absence of thinning of the retinal nerve layer at the time of diagnosis, we consider that the duration of optic nerve compression was short, contributing to the resolution of the visual disturbance. While a prospective study of prolactinoma patients who started cabergoline with 0.25-0.5 mg twice weekly reported marked resolution of visual defects up to three months [20], we could achieve sufficient improvement with a lower dose (0.25-0.5 mg once weekly) of cabergoline. As a long-term effect of cabergoline, secondary visual field deterioration due to chiasmal herniation [14] has been reported. We will continue to closely monitor the patient in the outpatient clinic, paying attention to the change in visual acuity in association with the prolactin level.

## Conclusions

We report a case of macroprolactinoma with a resolution of visual disturbance. In this case, serial ophthalmological exams including the measurement of visual fields, CFF, and OCT contributed to predicting the resolution of the disturbance in vision. These results were instrumental in predicting the visual outcome in accord with the pharmacological treatment. Seeking immediate medical attention after the onset of visual disturbance and optical assessment at the time of diagnosis may contribute to the restoration of visual acuity with no impairment of the prognosis.
